# Outbreaks in the Neonatal Intensive Care Unit: Description and Management

**DOI:** 10.3390/tropicalmed9090212

**Published:** 2024-09-12

**Authors:** Chryssoula Tzialla, Alberto Berardi, Vito Mondì, on behalf of the Study Group of Neonatal Infectious Diseases

**Affiliations:** 1 Neonatal and Pediatric Unit, Polo Ospedaliero Oltrepò, ASST Pavia, 27100 Pavia, Italy; 2 Neonatal Intensive Care Unit, University Hospital of Modena, 41124 Modena, Italy; alberto.berardi@unimore.it; 3 Neonatology and Neonatal Intensive Care Unit, Policlinico Casilino, 00169 Rome, Italy; vito.mondi@mail.com

**Keywords:** outbreak, neonate, NICU, healthcare-associated infections

## Abstract

Healthcare settings, especially intensive care units, can provide an ideal environment for the transmission of pathogens and the onset of outbreaks. Many factors can contribute to the onset of an epidemic in a neonatal intensive care unit (NICU), including neonates’ vulnerability to healthcare-associated infections, especially for those born preterm; facility design; frequent invasive procedures; and frequent contact with healthcare personnel. Outbreaks in NICUs are one of the most relevant problems because they are often caused by multidrug-resistant organisms associated with increased mortality and morbidity. The prompt identification of an outbreak, the subsequent investigation to identify the source of infection, the risk factors, the reinforcement of routine infection control measures, and the implementation of additional control measures are essential elements to contain an epidemic.

## 1. Introduction

Neonates admitted to neonatal intensive care units (NICUs), especially those born preterm, are particularly vulnerable to healthcare-associated infections (HAIs). Neonates are particularly susceptible to infections due to poor cutaneous and mucosal barrier mechanisms, underlying critical illness, poor macrophage function, poor opsonization, and low levels of serum immunoglobulins and complement [1,2,3]. Neonates admitted to NICUs are more exposed to pathogens [4,5], with some being resistant to antibiotics, due to the need for frequent invasive procedures, surgical interventions, prolonged hospital stays, and frequent contact with healthcare personnel.

Surveillance studies report that rates of HAIs in NICUs range from 8.7% to 74.3% [6]. In neonates with a gestational age of less than 32 weeks and a birth weight of less than 1500 g, the HAI rates range from 5.6% to 34.4% within the first 120 days of life and are inversely proportional to gestational age and birth weight [7]. In the recent point prevalence survey of healthcare-associated infections and antimicrobial use in European acute care hospitals carried out between 2022 and 2023, the adjusted OR for patients at risk for HAI was 1.4 and 1.6 for patients with an age of less than 1 month and with a birth weight of less than 1500 g, respectively [8]. HAIs are associated with high rates of mortality and morbidity and adverse neurodevelopmental outcomes, and they lead to increased healthcare costs [4,9,10]. The most prevalent forms of neonatal HAIs, particularly among very low birth weight infants, are central line-associated bloodstream infections (CLABSI) with an incidence range of 3.2 to 21.8 per 1000 central venous catheters days [4,5,11] and ventilator-associated pneumonia with an incidence rate reported to be between 2.7 and 37.2 per 1000 ventilator days [4,11,12]. A recent retrospective cohort study of over 300 NICUs in the United States reported a rate of hospital-onset bacteremia (HOB), a new surveillance measure for HAIs introduced by the Centers for Disease Control and Prevention (CDC), of 1.1 per 1000 patient days. Notably, the authors highlighted that 55% of the HOB events occurred in infants without a central line [13]. In the NICUs, more than half of the HAIs were found to be hospital-acquired sepsis [14].

Other forms of HAIs in neonates include catheter-associated urinary tract infections, central nervous system infections, skin and soft tissue infections mostly surgical site infections or eye infections. The reported rates of these infections vary considerably [9,10,15].

NICUs are particularly high-risk settings for the occurrence of epidemic events. An outbreak is generally defined as an unexpectedly high or otherwise excessive number of disease cases in a particular area, group of people, or care setting within a specific time period [16,17]. Identifying an outbreak depends on the normal frequency of that disease; the increase in cases of a particular infection must be statistically significant compared to the usual incidence for that area, population, and season. According to the World Health Organization (WHO), an epidemic is an outbreak of a disease that spreads quickly to more people than normally expected and moves into a large geographic area [18]. The definition of an epidemic is very similar to the definition of an outbreak, and some health organizations have the same definition for both terms. An epidemic/outbreak presents a geographically limited spread of disease, whereas a pandemic is characterized by the widespread transmission of a disease across a vast geographical area, often on a global or continental scale [18].

An epidemic event can result from an increase in the infectiousness of an already endemic disease or from the spread of infection from other geographical areas. The number of cases indicating an epidemic is contingent upon the pathogen in question, the type of population affected, and the time and place of occurrence. The disease may be a novel occurrence or a previously documented phenomenon within the given context. An epidemic event can occur over a relatively short or prolonged period (years) and affect a clearly defined group of patients or involve multiple hospital departments or even different hospitals [19].

Specific situations may serve as warning triggers, such as the identification of a multidrug-resistant microorganism isolated in a single patient admitted to a facility with highly vulnerable patients, which has never been identified in that specific environment [20]; the colonization of three or more neonates by the same Gram-negative bacterium; or a single case of systemic infection by extended-spectrum beta-lactamase (ESBL) or carbapenemase-producing *Enterobacteriaceae* (CRE) (resistant pathogens with a high risk of therapeutic failure) or *Pseudomonas aeruginosa* (the presence of which indicates a possible environmental reservoir) [21].

Outbreaks represent a serious healthcare problem because of the following [22]:

- They frequently occur in high-risk departments;
- Responsible microorganisms are often resistant to common therapies;
- They are associated with high mortality rates;
- They are sometimes related to errors in care practices;
- They highlight often unknown or underestimated care issues.

## 2. The “Anatomy” of an Outbreak and Its Management

The transmission of infectious agents in healthcare settings requires the presence of a source (or reservoir) of infectious agents, a susceptible host, and a mode of transmission.

### 2.1. Risk Factors

In NICUs, various factors increase the risk of infection for a neonate, including the following [20,21,22,23,24]:

An immature immune system;

- The presence of comorbidities;
- The need for various invasive procedures (mechanical ventilation, the use of a central catheter and enteral tubes, etc.);
- A high frequency of contact with personnel;
- Inappropriate/prolonged use of antibiotics;
- A prolonged hospital stay;
- Non-compliance with hygiene standards;
- Overcrowding in the ward;
- Structural or equipment deficiencies;
- An inadequate staff-to-patient ratio.

### 2.2. Pathogens Responsible for Outbreaks

Any infectious agent transmitted in healthcare settings may be epidemiologically significant under certain conditions. The CDC [25] defines epidemiologically significant pathogens as those with the following characteristics:

(a) Microorganisms with a propensity for transmission within healthcare facilities (e.g., *Clostridium difficile*, norovirus, respiratory syncytial virus, influenza virus, rotavirus, *Enterobacter* spp., and *Serratia* spp.);
(b) Microorganisms that are resistant to first-line therapies;
(c) Microorganisms that are difficult to treat due to their resistance to multiple classes of antimicrobials;
(d) Both common and rare microorganisms with unusual resistance patterns within a healthcare facility;
(e) New or re-emerging pathogens.

A 2017 literature review [26] identified that bacteria are the most frequently responsible for epidemics (76.9% of cases) in the neonatal period, particularly *Staphylococcus aureus*, *Klebsiella pneumoniae*, and *Serratia marcescens*, followed by viruses (12.8% of cases), fungi (7.7% of cases), and protozoa (2.56%). Additionally, approximately 57% of Gram-negative pathogens and 55% of Gram-positive pathogens were reported to be multidrug-resistant.

More recent data on neonatal outbreaks can be obtained from the Outbreak Database, a free accessible global database of healthcare-associated outbreaks [27]. We carried out the database search for this review on 30 June 2024 [28] to investigate the reported outbreaks, making queries through the “advance search” function using the keyword “newborn” in the “outbreak/setting/age” field. The items studied included causative pathogens, the type of facility, sources of the outbreaks, and measures taken to stop the outbreaks.

**Figure 1:**
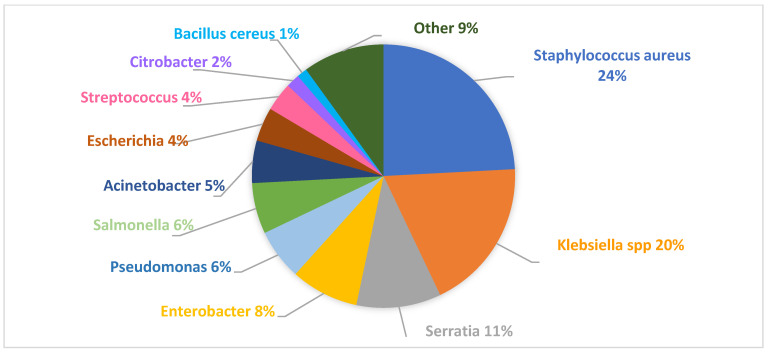
Pathogens that most frequently cause outbreaks in the neonatal period.

The outbreak database contained a total of 530 epidemic events. Of these, 82% were due to bacterial agents, 12% to viruses, and 6% to fungi. Among the bacterial outbreaks contents in the database, 65% were caused by Gram-negative pathogens and 35% by Gram-positive pathogens. Notably, the most frequently reported microorganisms were S. aureus (in 24% of cases) and *Klebsiella* spp. (in 20% of cases) (Figure 1) [28]. The epidemiological situation differs when considering the bacteria that most frequently cause epidemics in the NICU. In this setting, *Klebsiella* spp. is the pathogen most often responsible for epidemics (in 21.6% of cases), followed by *S. aureus*, which is responsible for 19.6% of cases [28].

### 2.3. Modes of Pathogen Transmission, the Identification of the Infection Source, and the Recognition of an Outbreak

Transmission modes vary depending on the type of microorganism, with some pathogens being transmitted through multiple routes. The main modes of transmission are the following [20]:

(a) Contact transmission: This is the most common mode and can be divided into two categories:
  - Direct contact (the transmission of the pathogen from an infected person to another person without an intermediary object or a contaminated person).
  - Indirect contact (the transfer of an infectious agent through an intermediary object or a contaminated person, e.g., via the hands of healthcare personnel when proper hand hygiene is not performed or with devices or instruments used for patient care that are not adequately sanitized between patients).
(b) Droplet transmission (through a short-distance airborne route).
(c) Airborne transmission (from patients or the environment): This occurs with pathogens that remain infectious for a long time and over distances. Microorganisms transported this way can be dispersed over long distances by air currents and can be inhaled by susceptible individuals even if they have not had direct contact with the contagious individual.
(d) Other sources of infection: These are associated with common environmental sources or vehicles (e.g., contaminated food, water, or medications).

Multiple infection sources are associated with epidemic events. Infectious agents transmitted in healthcare settings are primarily derived from human sources, although they can also involve environmental sources [19]. The source of infection varies according to the pathogen involved and often coincides with the reservoir, defined as the place where a particular microorganism can survive and multiply [16,17,19]. Human reservoirs include patients, healthcare personnel, family members, and/or other visitors. Individuals acting as sources/reservoirs may have active infections, be asymptomatic and/or in the incubation phase of an infectious disease, or may be transiently or chronically colonized by pathogenic microorganisms, particularly in the respiratory and gastrointestinal tracts [16,17,19].

The literature reports that the most commonly identified source during an epidemic coincides with the index case, but it is often not identified [26,29]. Outbreak database research has shown that the infection source was the index patient in 19% of cases, but it was unknown in 51% [28].

To implement adequate and effective epidemic control measures, it is essential to ascertain both the reservoir and the infection source. In the case of a point source, susceptible patients are exposed almost simultaneously to the source, with a rapid increase in cases. Conversely, in instances of continuous person-to-person transmission, there is a slower increase in cases and it is not attributable to a common source. The secondary spread of the pathogen from person to person can even occur with a common exposure source [16].

The recognition of a probable outbreak can derive from microbiology/virology data of clinical cases of infections confirmed by positive cultures or by microbiological surveillance cultures performed in the unit [17]. Surveillance represents a fundamental tool for identifying single cases or groups of patients infected or colonized by epidemiologically important microorganisms [25]. It can be divided into “passive” surveillance, which is based on culture results obtained during clinical practice, or “active” surveillance, which is based on swabs performed on all hospitalized patients to identify asymptomatic colonized patients. For the most effective use of resources, targeted surveillance for the highest-risk areas or patients could be preferred over facility-wide surveillance, but multidrug-resistant organisms (MDROs) may require facility-wide surveillance [17,25].

The role of active surveillance cultures (ASCs) in preventing the spread of MDROs in the endemic setting is debated in the literature [30,31,32]. The European Society of Clinical Microbiology and Infectious Diseases (ESCMID) suggests that ASCs should be used as an additional measure and not as a routine test in the endemic setting [33]. The CDC states that, even if controlled trials have not been conducted to determine which cases could benefit more from ASCs, their use could be considered in some settings with vulnerable patients, particularly if other control measures failed to control transmission [25]. Regarding CRE, the WHO affirmed in their 2017 guidelines [34] that information regarding a patient’s colonization status does not constitute routine standard of care but should be known during an outbreak or in situations with a high risk of CRE acquisition, like possible contact with a patient colonized/infected with CRE or an endemic CRE prevalence. In an Italian position paper on CRE infections, the authors indicate that active screening is required in high-burden settings for all patients at risk of invasive disease if colonized upon admission and weekly during hospital stay [35]. A recent systematic review [36] showed a decline in HAIs due to CRE after implementing ASCs, even if it was not possible to determine the independent effect of active screening since it was included in the bundle of interventions. 

Concerning the neonatal population, a recent review and meta-analysis [37] demonstrated that routine screening for Gram-negative pathogens is not supported by the data even though the authors showed a correlation between colonization and risk of bloodstream infection. Another systematic review [38] showed limited evidence of the effectiveness of routine screening for Gram-negative pathogens in predicting sepsis. The UK’s current guidelines do not recommend routine screening for neonates to identify colonization from ESBL-producing *Enterobacteriaceae* [29]. However, for CRE, active screening seems to help prevent infection and future outbreaks [39,40,41,42].

### 2.4. The Management of an Outbreak

The evaluation and management of epidemic events can be complex, frequently requiring multiple actions to be carried out almost simultaneously. To prevent the spread of infection, it is essential for the investigation of an epidemic event to be both effective and systematic.

Upon identifying the existence of an ongoing epidemic event, it is necessary to promptly report it to the hospital committee for infection control and prevention and to the healthcare staff of the operational unit [19,43]. The ongoing epidemic event must also be communicated to all other NICUs operating in the same territorial area and to the parents of the hospitalized newborns [43].

Subsequently, the following steps are recommended [43,44]:

(a) Implement all standard precautions for the prevention of HAIs;
(b) Conduct an epidemiological investigation to characterize the microorganism, define its biological characteristics and susceptibility to antimicrobial drugs, identify the origin and reservoir, trace the transmission routes, and identify possible risk factors;
(c) Implement additional or specific measures, such as educating staff and parents, modifying care practices, adjusting parental access, ensuring compliance with space requirements to avoid overcrowding, and limiting admissions/closing the ward.

Important additional measures [20,21,25] that are not considered primary components of programs to prevent the transmission of infectious agents but enhance their effectiveness include the following:

- Implementing antibiotic stewardship programs [45,46];
- Carrying out post-exposure prophylaxis with antiviral agents [47];
- Using vaccines for both pre- and post-exposure prevention;
- Screening and limiting visitors with signs of transmissible infections.

Targeted decolonization to eradicate *S. aureus* in colonized newborns should be considered in addition to implementing standard prevention measures during an *S. aureus* epidemic event [48]. The optimal agent for decolonization in newborns has not yet been determined, but the use of intranasal mupirocin twice daily for 5–7 days is considered an acceptable choice [48,49]. However, the ESMICD does not recommend routine decolonization for multidrug-resistant Gram-negative pathogens [50].

Standard precautions for infection prevention in healthcare settings include the following [20]:

- Hand hygiene [51,52].
- The use of personal protective equipment (PPE). PPE refers to various devices used alone or in combination to protect mucous membranes, respiratory tracts, skin, and clothing from contact with infectious agents. The choice of PPE is based on the nature of patient interaction and/or the pathogen’s likely modes of transmission.
- Environmental control through cleaning and disinfecting surfaces with appropriate products based on the microorganism (during epidemic events, more frequent cleaning/disinfection may be necessary compared to standards) [53,54,55,56].
- The management of patient care equipment/devices. Equipment and medical instruments/devices should be cleaned and maintained according to the manufacturer’s instructions.
- The management of linens. Contaminated fabrics, including linens and patient clothing, can harbor pathogenic microorganisms. However, the risk of disease transmission is negligible if handled, transported, and washed safely.
- The placement of patients based on the mode of pathogen transmission, e.g., using single rooms, possibly with negative pressure, for patients with airborne infections.
- Respiratory hygiene/cough etiquette. This strategy, which became part of standard precautions after the SARS-CoV-2 pandemic, targets patients and accompanying family members with undiagnosed transmissible respiratory infections and applies to anyone showing signs of respiratory illness when entering a healthcare facility.

The isolation and cohorting of newborns who are infected/colonized with the same microorganism must be maintained until their discharge from the unit [57]. Staff should be organized into groups dedicated to specific cohorts, and the number of people (staff and visitors) accessing the isolation/cohort rooms should be minimized [43,48].

In departments without an active microbiological surveillance program, one should be activated immediately upon the declaration of an epidemic event. In such situations, the increased incidence of infection caused by a specific pathogen is accompanied by an increased rate of colonization among other hospitalized patients. Colonization serves as a reservoir for pathogen transmission as 42% of colonized newborns will develop an infection [58].

Microbiological surveillance during an epidemic should be carried out as follows [43,48,49,53,58]:

- For all inpatients from the date of the event’s onset or upon admission to the ward and then periodically until the event is eradicated;
- At the environmental level, including for surfaces and equipment (depending on the type of microorganism, such as *Pseudomonas*, *Acinetobacter*, *Serratia*, etc.);
- For staff (depending on the type of microorganism or if epidemiologically implicated in the microorganism’s transmission).

Additionally, during the epidemiological investigation of the epidemic event, molecular typing of the isolated bacterial strains must be performed, and their preservation must be ensured at the microbiology laboratory [19]. Molecular typing is carried out based on various methods. Pulsed-field gel electrophoresis is considered the gold standard [19] but requires analytical expertise [26]. PCR-based techniques (such as random amplification of polymorphic DNA or repetitive-element PCR) [19] have recently been used as they are relatively easy to perform and quick [59,60]. Whole-genome sequencing is one of the newest techniques used in ongoing epidemic investigations [61,62,63,64], allowing for strains responsible for the event and “background” strains to be distinguished. It offers acceptable costs and a rapid turnaround time.

In cases where the implemented measures have not been sufficient to contain the epidemic, the facility lacks enough rooms for isolation/cohorting, and/or there are a lot of patients and/or staff shortages are among the possible causes of the epidemic, restricting admissions and even closing the ward should be considered [21,43,44].

**Figure 2:**
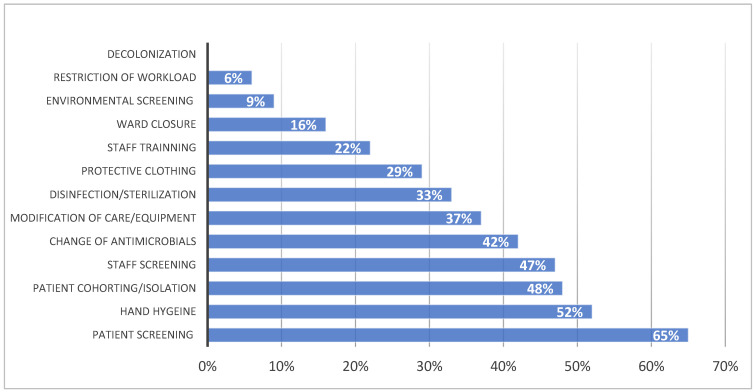
Measures implemented to control epidemic and frequency. Data source “Outbreak database” [28].

In most of the outbreaks highlighted by the outbreak database research, multiple control measures were adopted [28]. Patient screening, isolating or cohorting patients, enforcing hand hygiene measures, personnel screening, modifying care or equipment, and changing antimicrobic therapy were the most frequently introduced measures to contain epidemics among the outbreaks in the database (Figure 2). In 16% of database cases, the involved wards had to be closed to stop the epidemic [28].

### 2.5. Special Issues: Prevention of Central Line-Associated Bloodstream Infections (CLABSIs)

The use of invasive devices, such as a central intravenous line, is a risk factor for HAIs among neonates, and CLASBIs are common infections among NICU patients, causing considerable mortality and morbidity, prolonged hospitalization, and increased healthcare costs [5,65,66,67]. The extensive use of indwelling catheters, prolonged parenteral nutrition and/or medication administration, and frequent catheter manipulation in critically ill infants are some causes of the high prevalence of bloodstream infections (BSIs) [67]. The targeted surveillance of BSIs in high-risk patient areas like NICUs is an important step for the investigation and management of an outbreak [17,19]. Consequentially, CLABSI-preventing measures are included in infection control strategies [43]. CLABSIs are largely a result of poor technique at the time of placement and in the ongoing care of the catheter site; therefore, appropriate insertion and maintenance care bundles for central lines can effectively reduce the incidence of CLABSIs in the NICU [11,68,69,70].

A guideline from the CDC [66] and a paper from the white series of the Society for Healthcare Epidemiology of America (SHEA) [67] provide evidence-based recommendations for CLABSI prevention in the NICU for non-outbreak settings.

The choice of an appropriate catheter, site, and antiseptic agent according to the patient’s age, proper hand hygiene before insertion, and maximum sterile barrier precaution are the primary procedures to adopt when inserting a central line [66,67].

Specific procedures must also be followed during the management of the catheter after it has been placed, like only assessing the central line with sterile devices, maintaining an aseptic technique during manipulation, assessing the dressing’s integrity and catheter insertion site, performing appropriate central line dressing/infusion set change when required/according to the type of infusion, and assessing the need to maintain the central line daily [66,67].

Both the CDC and SHEA recommend against using prophylactic antibiotics during dwell time or when removing the central line [66,67].

## 3. Conclusions

Epidemic events in NICUs are associated with significant increases in morbidity and mortality among hospitalized newborns, both due to the intrinsic characteristics of the hospitalized patients and the type of microorganisms often involved. The early identification of the epidemic event, conducting an investigation and implementing standard prevention strategies to control HAIs, and activating additional specific measures are fundamental elements for containing and eradicating the epidemic.

## Data Availability

The data presented in this study are available in Outbreak database. at http://www.outbreak-database.com, accessed on 9 September 2024.

## Appendix A. Members of the Study Group of Neonatal Infectious Diseases

Agriesti Giovanni, Auriti Cinzia, Azzalli Milena, Baroni Lorenza, Bedetta Manuela, Berardi Alberto, Betta Pasqua, Bolognesi Serenella, Borghesi Alessandro, Brusa Giacomo, Busolini Eva, Capretti Maria Grazia, Carcione Angelo, Cassone Raffaella, Cavalleri Valeria, Cavigioli Francesco, Cecchini Enisia, Ciccia Matilde, Corona Maria Francesca, Cota Francesco, Crippa Beatrice, D’Alessandro Maria Carmelina, Dall’Agnola Alberto, Damiano Floriana, D’Andrea Vito, De Rose Domenico, De Filippis Raffaella, Della Casa Muttini Elisa, Di Comite Amelia, Di Dio Giovanna, Di Ricco Laura, Di Tommaso Eleonora, Fasolato Valeria, Fattori Ermanna, Fatuzzo Valentina, Floris Susanna, Frangella Emma, Gambini Lucia, Gasparrini Enrico, Giuffrè Mario, Gottardi Genny, Infriccioli Giovanna, Intini Angela, Lacerenza Serafina, Latorre Giuseppe, Lucente Maria, Magaldi Rosario, Mainini Nicoletta, Maino Marzia, Mancuso Domenica, Manzoni Paolo, Maragliano Giovanna, Marzollo Roberto, Mascheroni Donatella, Mastromattei Stefania, Mastricci Nunzia, Mazzeo Danila, Menonna Nicola, Menzato Federica, Migliaro Fiorella, Mondì Vito, Morandi Grazia, Nanni Francesca, Napolitano Marcello, Nardella Giovanna, Naselli Aldo, Natale Fabio, Notarmuzi Maria Letizia, Paolillo Piermichele, Pedicino Roberto, Perniciaro Simona, Perrini Stefania, Picone Simonetta, Pietrasanta Carlo, Pietravalle Andrea, Pivetti Valentina, Poggi Chiara, Porta Alessandro, Priante Elena, Proto Alice, Pugni Lorenza, Ridolfi Livia, Ronchetti Maria Paola, Ronchi Andrea, Salomè Serena, Serafini Lisa, Simonetti Debora, Sinelli Maria Teresa, Spadavecchia Alessia, Spanedda Giuseppina, Spinoni Vania, Stroppiana Paola, Tirrito Maria Ilaria, Tina Lucia, Torriero Roberto, Tzialla Chryssoula, Valastro Chiara, Valentino Liliana, Varalda Alessia.
